# Supplementary material for: A rock-salt-type Li-based oxide, Li3Ni2RuO6, exhibiting a chaotic ferrimagnetism with cluster spin-glass dynamics and thermally frozen charge carriers
Source: Sci Rep. 2016 Aug 22;6:31883. doi: 10.1038/srep31883 (PMC4992954; doi:10.1038/srep31883)
Supplement: Supplementary Information [file srep31883-s1.docx]

**Supplementary Information**

**A rock-salt-type Li-based oxide, Li_3_Ni_2_RuO_6,_ exhibiting a chaotic ferrimagnetism with cluster spin-glass dynamics and thermally frozen charge carriers**

**Sanjay Kumar Upadhyay,^1^ Kartik K Iyer,^1^ S. Rayaprol^2^, P.L. Paulose,^1^ and E.V. Sampathkumaran^1,*^**

^1^Tata Institute of Fundamental Research, Homi Bhabha Road, Colaba, Mumbai 400005, India

^2^UGC-DAE Consortium for Scientific Research, Mumbai Centre, R-5 Shed, BARC Campus, Trombay, Mumbai – 400085, India

*Corresponding author: [sampath@mailhost.tifr.res.in](mailto:sampath@mailhost.tifr.res.in)

Here, we show crystal structure of Li_3_Ni_2_RuO_6_ along the [100] direction (Fig. S1) and also compare raw neutron diffraction data for three different ranges in an expanded scale (Fig. S2) at three temperatures. In Fig. S1, we ignored crystallographic disorder discussed in the article, for the sake of simplicity.

Fig. S2 clearly brings out subtle variations in the intensity.


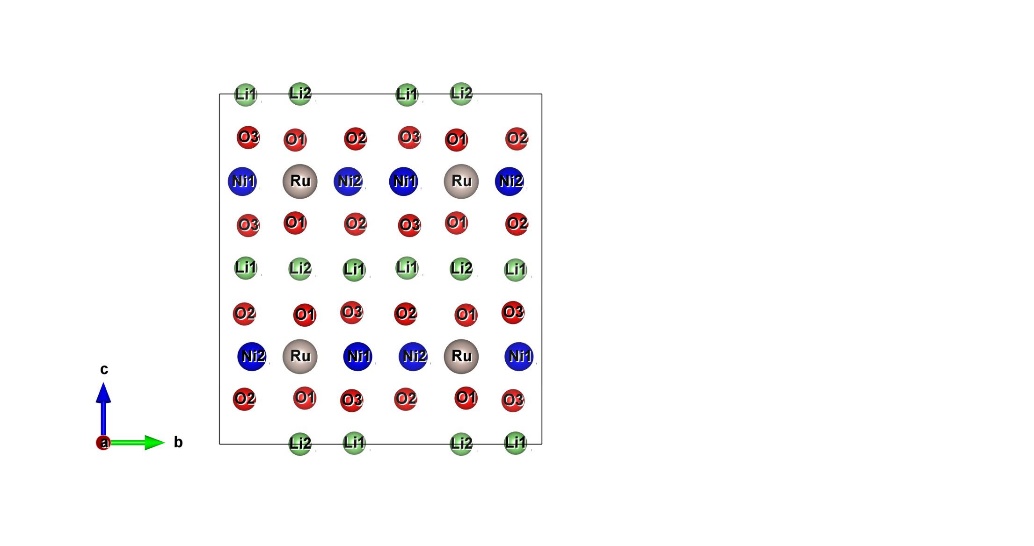


**Supplementary Figure S1:** Crystal structure of Li_3_Ni_2_RuO_6_ viewed along the [100] direction.


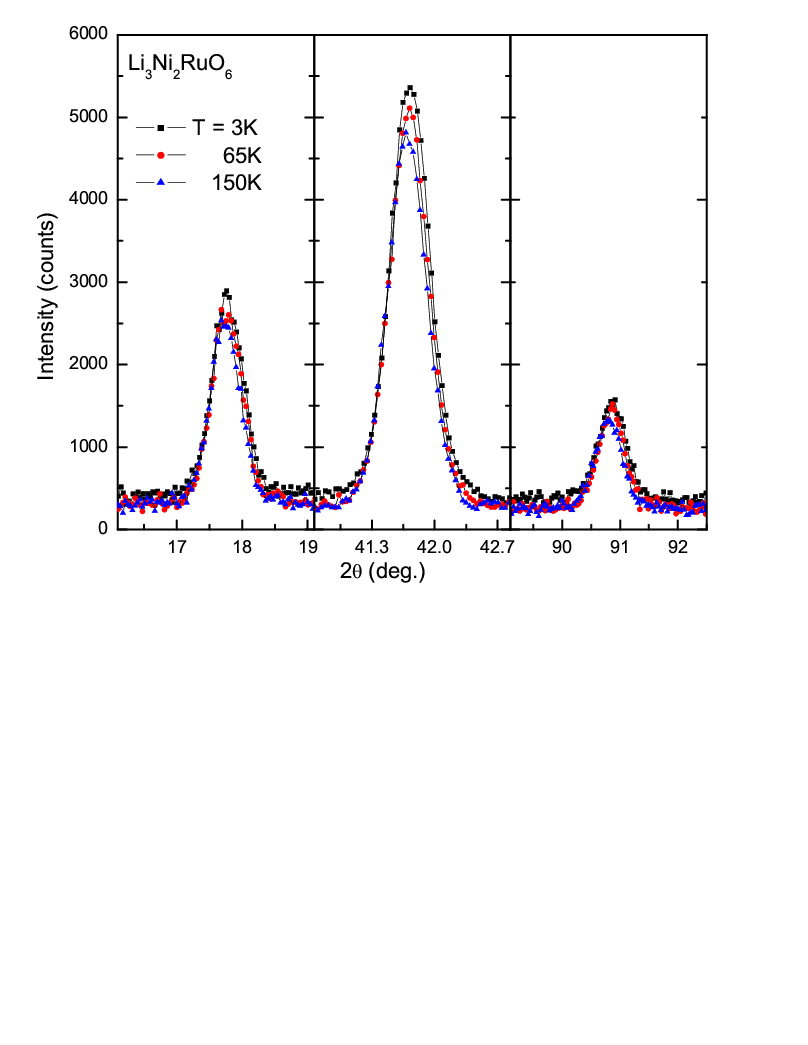


**Supplementary Figure S2:** The raw data of the neutron diffraction patterns recorded at T = 3, 65 and 150K are plotted on the same scale for three different regions.
